# False-positive troponin in a professional cyclist: a case report on avoiding misdiagnosis and unnecessary restrictions

**DOI:** 10.1093/ehjcr/ytag192

**Published:** 2026-03-10

**Authors:** Christophe Popelier, Koen Koppens, Wouter L’Hoyes, Alix Lambrecht, Tim Van Puyvelde

**Affiliations:** Hartcentrum Bonheiden Lier, Imeldalaan 9, Bonheiden 2820, Belgium; Department of Cardiovascular Diseases, KU Leuven, Herestraat 49, Leuven 3000, Belgium; Hartcentrum Bonheiden Lier, Imeldalaan 9, Bonheiden 2820, Belgium; Department of Cardiovascular Diseases, KU Leuven, Herestraat 49, Leuven 3000, Belgium; Hartcentrum Bonheiden Lier, Imeldalaan 9, Bonheiden 2820, Belgium; Hartcentrum Bonheiden Lier, Imeldalaan 9, Bonheiden 2820, Belgium; Hartcentrum Bonheiden Lier, Imeldalaan 9, Bonheiden 2820, Belgium; Department of Cardiovascular Diseases, KU Leuven, Herestraat 49, Leuven 3000, Belgium; Heart, Exercise and Research Trials (HEART) Lab, St.Vincent’s Institute of Medical Research, 9 Princes Street, Fitzroy, Victoria 3065, Australia

**Keywords:** Sports cardiology, Myocarditis, Cardiac troponin, Macrotroponin, Case report

## Abstract

**Background:**

Myocarditis can cause sudden cardiac death in athletes, and in the presence of clinical symptoms and elevated troponins, exercise restriction is recommended. However, macrotroponin complexes, formed by antitroponin antibodies, can cause falsely elevated troponin levels, thereby complicating the diagnostic process and leading to unnecessary exercise restriction.

**Case summary:**

A 27-year-old professional cyclist presented with exercise intolerance following a viral illness. High-sensitivity troponin I was markedly elevated, while high-sensitivity troponin T remained normal. Major cardiac causes were excluded, raising suspicion for macrotroponin complexes. This was confirmed via polyethylene glycol (PEG) precipitation. The patient was cleared to return to his sports activities without the need for further monitoring.

**Discussion:**

In athletes, exercise-induced troponin release is common and may contribute to a higher prevalence of macrotroponin formation, which is an underrecognized cause of elevated troponin levels. A discrepancy between troponin I and T assay results can suggest its presence, which can be confirmed using polyethylene glycol precipitation, a simple method that helps avoid unnecessary testing, activity restrictions, and prolonged monitoring.

Learning points

- Macrotroponin complexes should be considered when elevated troponin levels are present without clear pathology.
- A mismatch between elevated hs-TnI and normal hs-TnT strongly suggests assay interference. Polyethylene glycol (PEG) precipitation is a simple, cost-effective test to confirm the presence of macrotroponin complexes.

## Introduction

Myocarditis is a cause of sudden cardiac death in athletes, and intense exercise can aggravate myocardial inflammation and trigger ventricular arrhythmias.^1,2^ The diagnosis should be considered in athletes with suggestive symptoms and elevated troponin, as confirmed myocarditis requires exercise restriction for at least 1 month and until remission.^1^ A cardiac troponin value above the 99th percentile upper reference limit in the absence of signs or symptoms of myocardial ischaemia defines myocardial injury, and cardiac magnetic resonance (CMR) imaging should be considered to exclude myocarditis.^3^

However, while troponins are highly sensitive biomarkers of myocardial injury, they may occasionally be falsely elevated. One underrecognized cause is the presence of macrotroponin complexes.^4^ Recognizing this interference is essential to avoid unnecessary exercise restriction and misdiagnosis.

## Summary figure

**Figure:**
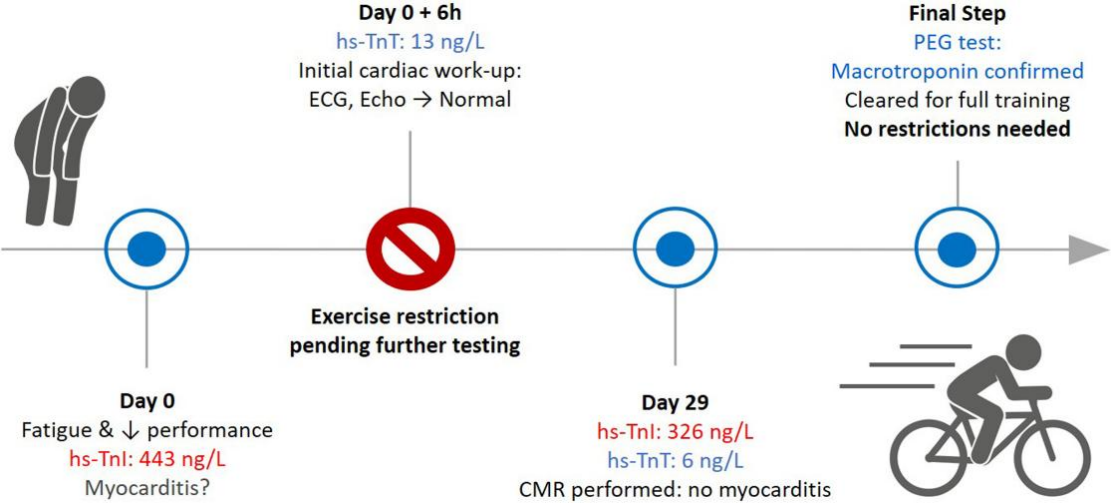


## Case presentation

A 27-year-old male professional cyclist was referred to the emergency department with a suspicion of myocarditis by his family doctor due to a 1-week history of fatigue, generalized myalgia, and elevated high-sensitivity troponin I (hs-TnI) (443 ng/l; normal value <34 ng/l).

Although he had no typical anginal chest pain or dyspnoea at rest, he noted increased exertional dyspnoea and reduced exercise tolerance during training sessions the past week. During intense exertion, he experienced a burning pulmonary sensation but no chest pain. He had experienced an episode of diarrhoea recently, but denied fever, dizziness, or syncope.

On presentation, the patient was afebrile and haemodynamically stable with a blood pressure of 128/69 mmHg and a pulse of 41 beats/min. Physical examination was within normal limitations. The patient had no significant medical history and he was a nonsmoker and denied consumption of illicit drugs, medication, or alcohol. He had been a competitive cyclist for the past 10 years, training approximately 20 h per week in recent years.

**Figure 1:**
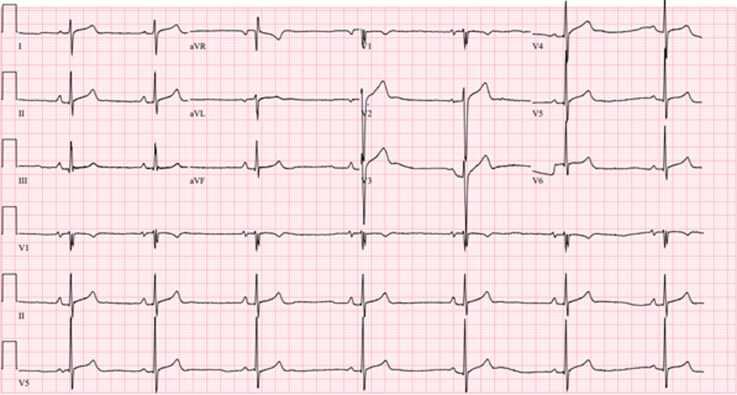
Electrocardiogram at presentation in the emergency room.

A 12-lead electrocardiogram showed sinus bradycardia with mild concave ST elevation consistent with an early repolarization pattern in V2 and V3, and fragmentation of the QRS complex in V1 (*Figure 1*). In trained athletes, the latter is a normal finding associated with an enlarged proximal right ventricular outflow tract.^5^ There was no PR segment depression suggestive of pericarditis. Chest X-ray revealed a normal cardiac silhouette with no evidence of pulmonary infiltrates or pleural effusion. A nasopharyngeal viral polymerase chain reaction test was performed and was negative for Influenza and SARS-CoV-2.

While hs-TnI had been significantly elevated when measured at an external laboratory, this elevation was not accompanied by an increase in creatine kinase (CK) or the creatine kinase-MB isoenzyme (CK-MB). When measured at the emergency department high-sensitivity troponin T (hs-TnT) repeatedly remained low, with values of 13 and 8.7 ng/l (normal <14 ng/l). Complete blood count, renal function, C-reactive protein, and coagulation parameters, including D-dimers, were normal.

Transthoracic echocardiography demonstrated normal biventricular systolic function and no wall motion abnormalities (see Supplementary material online, *Video S1*) (see Supplementary material online, *Table S1*).

Given the patient's clinical presentation and the initial findings, ambulatory Holter monitoring and CMR were planned for further evaluation. In the interim, the patient was advised to refrain from physical exercise.

**Figure 2:**
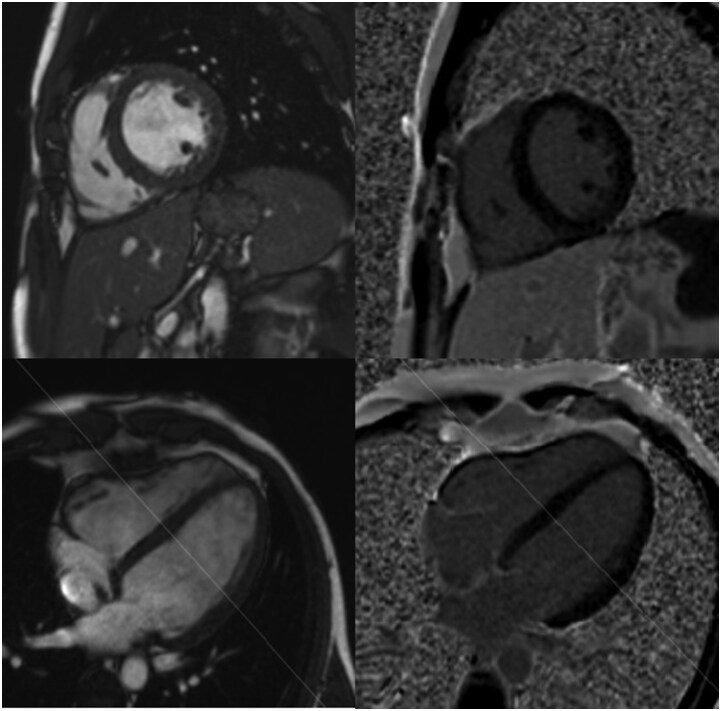
Cardiac magnetic resonance imaging showing absence of late gadolinium enhancement (top right: short axis; bottom right: 4-chamber) and presence of biventricular dilatation (top left: cine short axis; bottom left: cine 4-chamber).

Holter monitoring revealed a normal sinus rhythm with two isolated monomorphic premature ventricular contractions, consistent with an outflow tract origin (left bundle branch block pattern with inferior axis). CMR after 4 weeks revealed biventricular dilatation, consistent with physiological cardiac remodelling in an endurance athlete (‘athlete’s heart’) and preserved systolic function (see Supplementary material online, *Table S1*). No signs of focal or diffuse myocardial fibrosis or inflammation were observed on T2-weighted imaging, parametric mapping, or late gadolinium enhancement imaging (*Figure 2*). There was no pericardial effusion, nor were there signs of pericardial inflammation.

A repeat blood sample was obtained at the time of the CMR. While hs-TnT was again low (6 ng/l; normal <14 ng/l), hs-TnI remained markedly elevated (326.1 ng/l; normal <34 ng/l) (*Table 1*). Given this discrepancy, polyethylene glycol (PEG) precipitation was performed. PEG was added to the serum sample in a 1:1 ratio, followed by centrifugation.

**Table 1:** High-sensitivity troponin and CK values at presentation (Day 0), after referral to the emergency department (Day 0 +6 h) and during follow up (Day +29)

	Day 0	Day 0 +6 h	Day +29
High-sensitivity troponin I (ng/l, <34)	**443**	NA	**326**
High-sensitivity troponin T (ng/l, <14)	NA	13	6
CK-MB (ng/ml, <5,2)	2,4	3,3	NA
CK (U/L, <190)	48	54	NA

Normal values are displayed in parentheses. Values above the upper limit of normal are in bold.

Abbreviations: NA, not available.

PEG precipitation was consistent with the presence of macrotroponin complexes, with a PEG-precipitable activity (PPA) of 81.2% (reference range: <62.5%, based on five control samples). This result indicated that a significant proportion of the detected troponin I was due to macrotroponin complexes, circulating immunocomplexes that can lead to falsely elevated hs-TnI values, thereby explaining the discrepancy between the hs-TnI and hs-TnT measurements.

At follow-up, the exercise capacity normalized and the patient had no further symptoms. Following reassuring CMR findings, a bicycle exercise test was performed and demonstrated no inducible arrhythmias. He was counselled regarding the benign nature of macrotroponin complexes and was cleared to resume high-level training.

## Discussion

We report an endurance athlete with falsely elevated troponin due to macrotroponin, mimicking myocarditis and leading to unnecessary exercise restriction.

In athletes, elevated troponin levels post-exercise are not uncommon and are often attributed to transient myocardial stress.^6–8^ However, when associated with clinical symptoms, further testing is warranted. A key differential diagnosis in our patient was myocarditis, particularly in the context of recent viral illness. CMR is the most useful diagnostic tool for myocarditis and pending the results, our patient was advised to refrain from physical exercise, as is consistent per current guidelines.^1,2,9^ Importantly, while the 2015 ESC Guidelines for the diagnosis and management of pericardial diseases advise a 6-month exercise restriction in athletes with myocardial involvement, this has been changed to a more individualized approach in the recent 2025 ESC Guidelines for the management of myocarditis and pericarditis, with exercise restriction for at least 1 month and until remission.^1^

However, CMR showed no evidence of myocardial inflammation according to the updated 2018 Lake Louis criteria and was completely normal for an endurance athlete. Although imaging was performed 4 weeks after symptom onset, when inflammation may have partially resolved, myocardial oedema would typically still be detectable on T1 mapping and, with greater specificity, on T2 mapping.^10^

The discrepancy between elevated hs-TnI and normal hs-TnT raised the suspicion of falsely-positive troponin levels. One underrecognized cause is the presence of macrotroponin complexes. These complexes are formed by endogenous antibodies, potentially triggered by a broad range of causes, including viral infections, cardiomyopathies, valvular disease, myocarditis.^4^ However, these antibodies can also form in healthy individuals and have been identified in up to 20% of the population.^11^ These complexes have an increased half-life and interfere with immunoassays by capturing both the capture and the detection antibody, giving rise to false positive results.^4^

For reasons that remain unclear, troponin I is more commonly affected than troponin T, with the prevalence of anti-TnI antibodies reported in 10%–20% of patients with cardiac disease, whereas only 2%–5% have circulating anti-TnT antibodies.^4^ As in our case, discrepancy between elevated hs-TnI and normal hs-TnT levels is thus an important indicator for the presence of macrotroponin complexes. To confirm, several techniques are available. While, no gold standard exist, PEG precipitation is a simple and low-cost method.^4^

Given the increasing use of high-sensitivity troponin assays in both clinical and screening settings, clinicians should be aware of macrotroponin interference. In athletes in particular, repetitive physiological stress can lead to transient troponin elevations, which has been suggested to contribute to the formation of antitroponin antibodies and macrotroponin complexes.^4,6–8^

Early consideration of macrotroponin testing in appropriate clinical contexts can streamline workup, reduce patient anxiety, and, in athletes, allow for a safe and timely return to physical activity.

## Supplementary Material

ytag192_Supplementary_Data

## Data Availability

The data underlying this article are available in the article and in its online Supplementary material.

## Lead author biography

Koen Koppens, MD, is a cardiology resident at KU Leuven with a clinical focus on acute, interventional, and structural cardiology. He is currently working at AZ St. Lucas in Ghent, and his background includes training across KU Leuven, Imelda Hospital, and Amphia Hospital. He aims to further develop his advanced procedural skills and to pursue a career in interventional cardiology.
