# Supplementary material for: False-positive troponin in a professional cyclist: a case report on avoiding misdiagnosis and unnecessary restrictions
Source: Eur Heart J Case Rep. 2026 Mar 10;10(3):ytag192. doi: 10.1093/ehjcr/ytag192 (PMC13012598; doi:10.1093/ehjcr/ytag192)
Supplement: ytag192_Supplementary_Data [file ytag192_supplementary_data.zip › Supplementary table.pdf]

	TTE value	CMR value
LVEDd (mm)	53	59
LVEDV (mL)	NA	237
LVESV (mL)	NA	100
LVEF (%)	60	58
GLS (%)	-19.9	-17.6
MV E/A	2.45	NA
E/E’	5.6	NA
RVEDV (mL)	NA	261
RVESV (mL)	NA	130
RVEF (%)	NA	50
TAPSE (mm)	25	30
RVOT prox. diameter	35	NA
RVOT distal diameter	28	NA
TR Vmax (m/s)	2.4	NA
T1 (ms)	NA	910
T2 (ms)	NA	44
ECV (%)	NA	30

Supplementary table 1: Relevant transthoracic and cardiac magnetic resonance imaging values

TTE: transthoracic echocardiography; CMR: cardiac magnetic resonance: LVEDd: left ventricular end-diastolic diameter; LVEDV: left ventricular end-diastolic volume; LVESV: left ventricular end-systolic volume; LVEF: left ventricular ejection fraction; GLS: global longitudinal strain; MV: mitral valve; RVEDV: left ventricular end-diastolic volume; RVESV: right ventricular end-systolic volume; RVEF: right ventricular ejection fraction; TAPSE: tricuspid annular plane systolic excursion; RVOT: right ventricular outflow tract; TR: tricuspid regurgitation; ECV: extracellular volume
